# Novel Variants Affecting Plasma Interleukin-6 Among A Healthy Aging Population: The Long Life Family Study

**DOI:** 10.1093/geroni/igaf122.3819

**Published:** 2025-12-31

**Authors:** Mary Wojczynski, Lihua Wang, Bharat Thyagarajan, Kaare Christensen, Joseph Zmuda, Alexander Kulminski, Michael Province

**Affiliations:** Washington University School of Medicine, St. Louis, MO, Missouri, United States; Washington University School of Medicine, Saint Louis, Missouri, United States; University of Minnesota, Minneapolis, Minnesota, United States; University of Southern Denmark, Odense M, Syddanmark, Denmark; University of Pittsburgh, Pittsburgh, Pennsylvania, United States; DUKE University, Durham, North Carolina, United States; Washington University School of Medicine, Saint Louis, Missouri, United States

## Abstract

Aging is associated with chronic low-grade inflammation. Interleukin-6 (IL-6) is a pro-inflammatory cytokine, linked to many clinical conditions, and a marker of chronic inflammation. IL-6 increases with normal aging, and higher levels of IL-6 in older individuals have been associated with physical and cognitive impairment, as well as increased dementia risk. IL-6 may be a pathway to reduce chronic low-grade inflammation. We investigated if there were regions of the genome harboring protective IL-6 variants. We used data from the NIA Long Life Family Study (LLFS, N = 4,084), a multi-national, multigenerational study of healthy aging. Participants underwent a comprehensive in-home examination at baseline, including a blood draw. Plasma IL-6 (pIL-6) concentration and whole genome sequencing (WGS) was performed using blood samples. We completed a linkage analysis for pIL-6 (covariate adjusted) using SOLAR followed by association of WGS variants under the peak. Chromosome 22 at 8cM (21,830,838 base pairs) had a LOD of 2.77. A subset of 20 families, demonstrated a heterogeneity LOD score of 9.54. Association on these 20 families found eight variants with suggestive association (p < 1E-04), half protective. Via model selection, 5 variants (3 protective and 2 risk) remained in the model, collectively explaining 20% of the variation in pIL-6, and explaining 41.7% of the linkage. One variant was associated with an RNA transcript for the IGLV3-25 gene (p = 8.03E-09), which is a critical gene for the formation of functional antibodies produced by plasma cells. Further analyses of the association of these novel variants with healthy aging and related phenotypes is warranted.

